# The Missouri H5 flu case: a call for strengthening public health preparedness

**DOI:** 10.1097/MS9.0000000000002866

**Published:** 2025-01-09

**Authors:** Erum Siddiqui, Maliha Khalid, Muhammad Saad Khan, Aminath Waafira

**Affiliations:** a Department of Medicine, Jinnah Sindh Medical University, Karachi, Pakistan; b The Maldives National University, Malé, Maldives

The recent detection of human infection with an H5N1 (Hemagglutinin type 5 Neuraminidase type 1) avian influenza virus (bird flu) in the United States has reignited global concern about its potential as a major health threat. Since its first significant outbreak in 1996^[1]^, the highly pathogenic avian influenza (HPAI) virus has periodically resurfaced, causing substantial mortality in both avian populations and humans. The persistent occurrence of human infections, coupled with the virus’s high mortality rate, continues to underscore its potential to cause a major public health crisis.

## Epidemiological context

Globally, from 1 January 2003 to 27 September 2024, 904 human infections with the avian influenza A(H5N1) virus were documented across 24 countries, resulting in 464 deaths^[2]^. This translates to a case fatality rate of approximately 60%, a strikingly high figure that sets H5N1 apart from other zoonotic viruses in terms of its lethality^[3]^. Although the incidence of human infections remains low relative to the widespread circulation of the virus among birds, the unpredictable nature of influenza viruses, particularly their propensity for mutation and re assortment, raises the specter of a potential pandemic. The human cases of H5N1 infection have generally been linked to direct contact with infected birds or their secretions. However, recent reports from the United States have brought a new dimension to the issue. Since April 2024, CDC (Centers for Disease Control and Prevention) has confirmed avian influenza infections in 27 people in the United States. Nine of these cases were associated with exposure to H5N1^[4]^.

## Missouri case report

The human cases of H5N1 infection have generally been linked to direct contact with infected birds or their secretions^[5]^, but recent reports from the United States have brought a new dimension to the issue. On 6 September 2024, the Centers for Disease Control and Prevention (CDC) confirmed the 14th human case of H5N1 bird flu infection in Missouri, USA. This case is notable as it lacked the usual occupational exposure to infected animals. No ongoing transmission among close contacts or otherwise has been identified^[6]^. This development suggests that the virus may be altering its modes of transmission or adapting to new environmental or host conditions. The state of Missouri, where this case was reported, has not documented any significant outbreaks in its poultry industry, although infections have been confirmed in backyard flocks and wild birds. This case underscores the evolving nature of the virus and the importance of continuous vigilance and adaptation of our surveillance strategies.

## Avian influenza virus ecology

Avian influenza viruses, particularly those of the H5N1 subtype, are primarily viruses of birds. Wild waterfowl serve as natural reservoirs, and outbreaks in domestic poultry are common, particularly in densely populated agricultural regions^[7]^. However, the spillover into mammalian species, including humans, has long been a cause for concern. H5N1’s high pathogenicity in both avian and mammalian species, coupled with its potential for zoonotic transmission, makes it a prime candidate for close monitoring. In recent years, the global spread of H5N1 viruses, specifically those belonging to the clade 2.3.4.4b lineage, has garnered attention due to their enhanced transmissibility among wild birds and increasing adaptability to mammals^[8]^. The detection of mammalian adaptation markers in the viral genome indicates that the virus is evolving in ways that may increase its ability to infect humans, raising the prospect of more widespread human transmission. The possibility that H5N1 influenza could evolve into a virus capable of sustained human-to-human transmission is particularly troubling. Influenza viruses are well known for their ability to undergo antigenic drift and shift, resulting in the generation of novel viral strains with different immunogenic properties^[9]^. In the case of H5N1, a few genetic changes could potentially allow it to efficiently infect the human respiratory tract, facilitating human-to-human spread. If such an event occurs, the global public health impact could be devastating. Given the virus’s current case fatality rate of approximately 60%, even a modest increase in transmissibility could lead to a catastrophic outbreak.

## Insights from the recent H5N1 cases in the United States

The outbreak of human H5N1 cases in the United States in 2024, particularly the case in Missouri, presents a new challenge to public health authorities. Historically, human cases of avian influenza virus infection have been linked to occupational exposure to poultry or environmental exposure in areas where infected birds are present. The absence of such exposure in the Missouri case suggests that the virus may be adapting to new modes of transmission, although it is too early to draw definitive conclusions. Nonetheless, this case should serve as a wake-up call for public health officials and the scientific community to intensify their surveillance efforts and investigate possible routes of transmission that may have contributed to this infection. While the overall risk to the general population remains low^[6]^, the potential for the virus to acquire new transmission pathways poses a real and immediate danger. The potential for zoonotic influenza viruses to cause pandemics is well documented. The 1918 H1N1 influenza pandemic, which claimed an estimated 50 million lives worldwide, serves as a grim reminder of the consequences of a novel influenza virus adapting to human hosts^[10]^. Although H5N1 differs from H1N1 in its ecology and epidemiology but the lessons from past pandemics highlight the importance of preparedness and early intervention.

## Challenges in controlling H5N1 spread

One of the major challenges in controlling the spread of H5N1 is the virus’s widespread distribution in avian populations, particularly among migratory wild birds, which act as natural reservoirs^[11]^. Wild birds play a pivotal role in the global spread of H5N1, carrying the virus across continents during migration. The detection of H5N1 in wild bird populations in North America, Europe, Asia, and Africa underscores the global nature of the threat and the need for international cooperation in monitoring and controlling the virus^[12]^. The movement of wild birds over large distances and across different regions makes it nearly impossible to limit the virus geographically, which in turn increases the likelihood of spillover into domestic poultry and, ultimately, human populations.

Recent trends in H5N1 infections suggest that the virus is increasingly affecting urban populations, particularly as migratory bird routes intersect with densely populated areas, expanding the risk beyond traditional rural or agricultural settings. Climate change may exacerbate this, altering migratory patterns and enabling the virus to spread to new regions, while also influencing poultry practices and wild bird interactions. Additionally, underexplored risk factors, such as vulnerability in immunocompromised individuals or those with respiratory conditions, could lead to more severe outcomes if the virus evolves to be more transmissible. Furthermore, environmental reservoirs, like wetlands where wild birds congregate, may serve as hotspots for cross-species transmission, emphasizing the need for a broader ecological approach. Strengthening surveillance to include diverse human populations and utilizing emerging technologies, such as genomic sequencing and AI for early detection, is essential to address these evolving challenges. These insights highlight the need for adaptive strategies to better predict and mitigate the risk of H5N1 outbreaks.

Currently, the CDC utilizes the National Influenza Surveillance System, which includes both human and animal influenza tracking. This system integrates laboratory-based testing, genetic sequencing, and collaboration with global health organizations to monitor influenza variants and transmission trends. In addition, animal health programs focus on avian influenza in domestic and wild bird populations to identify zoonotic spillover risks early^[13]^. To strengthen these measures, an increase in geographic and environmental surveillance coverage is recommended, particularly in regions with high poultry farming activity or proximity to migratory bird paths. This enhanced approach could involve deploying real-time genetic analysis for faster detection of mutations indicative of heightened transmissibility or pathogenicity. Furthermore, incorporating wastewater surveillance in at-risk communities and improving inter-agency data sharing between human and veterinary health sectors would enhance early warning capabilities. By refining and expanding these existing frameworks, we can achieve a more comprehensive and responsive surveillance system, tailored to the evolving threat of H5N1.

## Strategies for preparedness

The One Health approach, which recognizes the interconnectedness of human, animal, and environmental health, is essential in tackling the threat posed by zoonotic diseases like H5N1^[14]^. This approach emphasizes the need for collaboration across multiple sectors, including public health, veterinary medicine, agriculture, and environmental science, to develop comprehensive strategies for disease prevention and control. In the context of H5N1, this means improving biosecurity measures in poultry farming, strengthening surveillance systems for both human and animal populations, and enhancing our understanding of the ecological factors that contribute to the spread of the virus.

A key component of H5N1 management strategy is the need for rapid and adaptable diagnostic tools for early detection and response to outbreaks. Ensuring that healthcare providers are well-trained in using these tools is critical for effective containment, and it also requires coordination of resources and expertise across borders. Vaccination remains one of the most effective tools in the fight against H5N1. In poultry populations, vaccination can significantly reduce the spread of the virus and prevent spillover into human populations^[15]^. However, vaccination campaigns have been hampered by logistical challenges, particularly in low- and middle-income countries where the resources needed to implement large-scale vaccination programs may be lacking. In countries where H5N1 is endemic, such as parts of Southeast Asia, vaccination efforts have had mixed results, with some successes in reducing the incidence of outbreaks but still facing challenges in achieving widespread coverage. Additionally, the rapid evolution of the virus complicates efforts to produce a vaccine that will remain effective over time. Continuous monitoring of viral mutations and incorporating these changes into vaccine formulations are necessary to ensure that vaccines remain effective. Effective public communication is crucial in managing outbreaks, requiring transparent, science-based messaging to maintain public trust and combat misinformation. Public health campaigns must promote awareness of risks from contact with wild birds and poultry, while encouraging better hygiene and biosecurity in agriculture. Antiviral drugs like oseltamivir (Tamiflu) show some effectiveness in treating H5N1 in humans, though challenges remain with timing and potential drug resistance. The recent H5 case in Missouri, especially with no known exposure to infected animals, highlights the need to reassess preparedness. Strengthening surveillance, enhancing biosecurity, and accelerating vaccine development are essential to prevent future outbreaks

## Conclusion

The recent H5N1 case in Missouri, especially with no known exposure to infected animals, highlights the need to reassess preparedness. Strengthening surveillance, enhancing biosecurity, and accelerating vaccine development are essential to prevent future outbreaks. The detection of H5N1 infection without clear exposure should serve as a critical reminder that viruses like H5N1 influenza are unpredictable and can adapt quickly. Public health preparedness must evolve accordingly. Through a combination of enhanced surveillance, diagnostic capabilities, vaccine development, and international cooperation, we can better position ourselves to respond to the next potential zoonotic outbreak. The lessons learned from this case in the United States should inform public health strategies moving forward, ensuring that we are not caught off guard by the next wave of H5N1 influenza.

## Data Availability

Not applicable.
